# Psychometric Analysis of the Spanish Version of the *Identity Distress Scale* in Ecuadorian Emerging Adults

**DOI:** 10.11621/pir.2024.0406

**Published:** 2024-12-15

**Authors:** Fernanda Cordero-Hermida, Juan Diego Sacoto, Evelyn Sinchi, Paúl Arias-Medina

**Affiliations:** a University of Cuenca, Ecuador; b Integral Psychopedagogy Center “Jade”, Cuenca, Ecuador

**Keywords:** identity distress, exploratory factor analysis, confirmatory factor analysis, construct validity, identity, emerging adulthood

## Abstract

**Background:**

Emerging adulthood is a newly known developmental stage in humans, between late adolescence and fully-achieved adulthood. This stage is characterized by continued indecision and postponement of individuation; it also corresponds with a delay in identity actualization. Personal identity is related to the development of the individual across various aspects of life including the identification of long-term goals, career selection, friendship, and sexuality. Therefore, identity is understood to be an individual’s perception of themselves and the conceptualization of their place within a social context. Identity development is expected to reach this critical stage between late adolescence and emerging adulthood, where specific factors may arise to hinder the realization of identity, potentially resulting in identity problems or identity distress. The latter considered a disorder within the DSM-III, and based on its diagnostic criteria, the *Identity Distress Scale* was created to measure the presence of identity distress.

**Objective:**

First, to report evidence of validity and reliability of the IDS using exploratory and confirmatory factor analysis, internal consistency, and invariance testing according to the Identity Distress Scale across a sample of Ecuadorian emerging adults. Second, to report the scores of the Identity Distress Scale among the participants.

**Design:**

The study employed a quantitative approach with a non-experimental, cross-sectional design, and an instrumental scope.

**Results:**

The results reveal that the scale operates with a first-order two-factor model, demonstrating a good fit and internal consistency (χ^2^(df) = (34) 125.03, CFI= .97, TLI = = .95, SRMR = .07, RMSEA = .07, α = from .72 to .76, ω = from .66 to .73). Evidence of measurement invariance was found between males and females.

**Conclusion:**

The IDS has adequate psychometric properties for its use in the Ecuadorian context.

## Introduction

Emerging adulthood (EA), a transitional developmental stage between late adolescence and early adulthood (Barrera-Herrera & Vinet, 2017), spans 17 to 29 years of age (Arnett, 2015; Wood et al., 2017). It signifies a time of continued development and self-discovery for individuals, who, in parallel with generational sociodemographic changes, are pursuing new experiences (Arnett, 2005; Gfellner & Córdoba, 2023).

According to Arnett, emerging adults tend to refrain from making commitments such as marriage or family planning. Instead, they are driven by the need to pursue various goals related to higher education, engagement in informal employment, pursuit of new experiences, and exploration of diverse romantic and sexual relationships. This inclination may lead emerging adults to engage in risky and harmful activities such as substance abuse, promiscuous and unsafe sexual behaviors, and even antisocial behaviors (Salvatore, 2018).

One of the main characteristics of EA is a phase of identity exploration (Arnett, 2000). In this exploratory stage, individuals are unsure about who they are, what they want, or what they seek in others (Salvatore, 2018). The formation of identity, a crucial concept for understanding the adolescent developmental process, has therefore extended to EA (Gfellner & Cordoba, 2017).

Within the field of psychology, identity was first explored through psychoanalytic theory. Although psychoanalytic literature does not explicitly use the term identity, it does refer to *identification*, to explain how conflicts arise between levels of consciousness and consequently influence the emergence of personality (Elgarte, 2011).

Freud (1923/1986) conceptualized the ego as the defensive pole of personality, responsible for mediating and directing impulses in response to the demands of reality. Along the same theoretical line, Lacan (1961/2009) conceptualized the process of identification as a foundational mechanism in the very constitution of an individual, regarding the subject’s relation to the symbolic. Conversely, Erikson (1985), who, elaborated on the concept, defined personal identity as “the perception of sameness and continuity of one’s existence in time and space and the perception that others recognize that sameness and continuity” (p.19).

According to Erikson’s theory, identity formation occurs throughout life, but during adolescence, the process reaches its most critical phase, the time when individuals seek to establish their place in the society they are immersed in (Tesouro, et al., 2013).

Therefore, the development of identity is related to what makes the individual different from others; it involves self-discovery, future goals, values, and beliefs, which in turn enables individuals to position themselves within a certain social group they feel capable of belonging to (Barrera-Herrera & Vinet, 2017; Tesouro et al., 2013). There are facilitating factors and factors that hinder identity development. Among the facilitating factors, are context and its diversity, the ability to make decisions, having a partner, and being independent which promotes identity formation. Conversely, factors such as dependence on parents, insecurity, and dependence on others’ opinions would limit identity achievement (Barrera-Herrera & Vinet, 2017).

Identity is primarily formed through social interaction across three dimensions: self-recognition, recognition of others, and others’ recognition of us (Marcús, 2011). Given that development through adolescence and EA requires stable mental health (Yuguero et al., 2020), instability caused by a deficit or delay in psychological maturity, can incur the risk of potential maladaptation and even possible mental health problems (Gfellner & Córdoba, 2017). Moreover, a strong characterological conflict can confuse the individuals in their perception of themselves (Erikson, 1985).

The difficulty in this transition from adolescents into adulthood is evidenced in resolving both the identity crisis (Erikson 1968, cited in Sica et al., 2014) and the conflict between identity synthesis and role confusion (Crocetti et al., 2012). Due to the different decisions and changes faced by adolescents, the subsequent EA stage is prone to increasing identity distress (Berman et al., 2004). Contemporary youth may exhibit a variety of identity distressers due to several factors including, but not limited to a prolonged period of academic study, and job uncertainty (Luo et al., 2020). Identity distress has been directly related to psychological instability and it is considered a significant issue among university students (Gfellner & Córdoba, 2020). Problems related to identity may even be considered pathological (Berman, 2020).

Within the study of identity, identity distress is a focus that has not been deeply investigated but has gained strength in recent years. Berman et al. (2004) developed the Identity Distress Scale (IDS) to study this phenomenon. This instrument is derived from the diagnostic criteria for identity disorder, included in the DSM-III and DSM-III-R, although this classification was modified in the DSM-IV (1994), where the disorder was reclassified as an identity problem and categorized among other conditions that may be a focus of clinical attention.

The main characteristic of this disorder, according to the DSM-III (1980), is the development of severe subjective distress caused by an inability to reconcile aspects of the self into a single coherent and acceptable sense of personhood. The diagnostic criteria evaluated in the IDS include the degree of long-term goals, career choice, friendships, sexual orientation and behavior, religious identification, moral value systems, and group loyalties.

In addition to these seven distress factors, the IDS also evaluates a *global* factor, which measures the generalized distressing effects of the aforementioned criteria on the participant’s life. Finally, item 10 of the IDS addresses the timeframe of discomfort, distress, or concern associated with the evaluated criteria. This last item fulfills the diagnostic criterion of symptoms across time, which, according to the DSM-III, must be present for at least three months (DSM-III, 1980).

The authors report acceptable values of internal consistency (α = .84), reliability (κ = .82), and convergent validity; the correlations were significant and ranged between r = .11 and r = .64). The survey is scored in the event a participant meets the diagnostic criteria for identity disorder; however, this scale has not been validated in Ecuador.

In terms of research, Capella & Andrade (2017) acknowledge that psychology in Ecuador lacks widespread dissemination of empirical studies, whether qualitative, or quantitative. Additionally, in Ecuador, the repertoire of psychometric instruments suitable for use in research is limited As Costales (2011) asserts, assessment tools used in Ecuadorian organizations (cognitive tests, personality inventories, scales, surveys, etc.) lack psychometric studies supporting their validity and effective application (p. 6).

The present study aims to evaluate the construct validity, internal consistency, and invariance of the IDS tool. Psychometric studies of the Spanish version of this instrument are nonexistent. According to the reviewed literature, the IDS is the only tool that addresses the construct of identity distress, applicable to various age ranges, but especially during EA. Finally, the present study contributes to an understanding of how identity develops in Ecuadorian emerging adults. The findings not only enhance theoretical knowledge but also provides a basis for practical interventions that can support emerging adults in their journey towards establishing an identity. Additionally, the study lays the foundation for future research into identity in Ecuador and the broader Latin context.

## Methods

The study adopted a quantitative approach with a non-experimental, cross-sectional design, and an instrumental scope.

### Participants

The sample consisted of 517 university students (67.9% female and 32.1% male). Additionally, the population fell within the age range of 17 to 29 years. The ages comprising the population correspond to the developmental stage known as emerging adulthood.

### Procedure

The instruments were administered to psychology students at the University of Cuenca after receiving approval from the dean. Initially, a pilot test was conducted with ten volunteers who reviewed the Spanish translation of the instruments and assessed the appropriateness of the content. Subsequently, participants were selected based on convenience sampling. Both the consent form and the application form were provided in digital format, and students were directed to a computer lab to complete the necessary documentation.

### Questionnaire

The Identity Distress Scale, developed by Berman et al. (2004), aims to measure severe interference or disturbance in identity development according to the conceptualization of identity distress (DSM-III) and identity problems (DSM-IV TR). It consists of 10 items, the first seven items are created based on a 5-point Likert scale response format (from 1 = not at all, to 5 = very severely) to indicate the extent to which respondents have been recently upset, distressed, or worried about identity issues: long-term goals, career choice, friendships, sexual orientation and behavior, religion, values and beliefs, and group loyalties. Two items prompt respondents to rate the overall level of discomfort, distress, or anxiety and how much uncertainty interferes with their life overall. One final item asks respondents to indicate the time (from 1 = never to 5 = more than 12 months) they felt upset, distressed, and worried about these issues.

#### Data Analysis

A qualitative review process was conducted which included translations from English to Spanish and back-translations from Spanish to English, as well as a qualitative evaluation of item wording and relevance to context and culture.

Quantitative analysis was conducted in three stages: the first stage involved descriptive analysis; the second stage comprised exploratory and confirmatory factor analyses and internal consistency analyses; the third stage involved invariance analysis and comparisons of scores across the categories of sex.

Skewness, discrimination, and difficulty values are reported. Discrimination, also referred to as item-total correlation, indicates how participants perform on the test as a whole compared to how they behave on each item individually. Values > .3 are considered good, values between .1 and .3 are acceptable, and values < .1 are poor (Mikulic, 2007). Regarding difficulty, this reflects how strongly respondents endorse higher-end responses to an item; high values indicate that respondents frequently select higher response categories, and those close to 0 indicate that respondents generally select lower response categories, with the ideal range between .5 and .8 (Aiken, 2003).

The Kaiser, Meyer, and Olkin (KMO) statistic is calculated with values ≥ .8 considered acceptable. Additionally, Bartlett’s sphericity test is performed, and a significant result is expected. Factor extraction is performed using the unweighted least squares method (ULS), as it is the most efficient for exploratory factor analysis (Lloret-Segura et al., 2014). The number of factors to be extracted is determined by utilizing very simple structure (VSS) and Velicer’s minimum average partial (MAP) analysis (Horn, 1965; Revelle, 2020).

Classic indices of absolute and incremental fit are used to evaluate the goodness of fit. The χ^2^/degrees of freedom ratio is expected to be less than 3, the RMSEA value and its confidence interval are expected to be less than .06, although other authors suggest a cutoffof .08 (Browne & Cudeck, 1989). The SRMR value is expected to be less than .08 (Steiger & Lind, 1980), and CFIand TLI values are expected to be above .95 (Hu & Bentler, 1999). Internal consistency of the instrument was evaluated using ordinal Cronbach’s alpha (α), and McDonald’s omega (ω) as suggested by Raykov (2001). Average variance extracted (AVEVAR) scores above .50 are considered to show adequate convergence across all factors (Franco-Guanilo & Hervias-Guerra, 2022). Scores between .7 and .8 are considered acceptable, values above .8 indicate high consistency, and values above .9 may indicate question redundancy (Cicchetti, 1994; Tavakol & Dennick, 2011). Invariance analysis begins by assessing population covariance equality, followed by configurational, metric, residual, and scalar invariance applying the criteria proposed by Cheung & Rensvold (2002).

Lastly, the behavior of the variables *age* and *sex* within the two-factor model is analyzed. Regarding age, its correlation with the distress and global factors is analyzed. Similarly, the sex variable is compared within both factors, but also subjected to distribution tests to verify the magnitude of the difference between males and females.

All analyses were conducted using R software (R Core Team, 2021) utilizing several packages including lavaan (Rosseel, 2012), nFactors (Raiche & Magis, 2020), psych (Revelle, 2020), sjPLot (Lüdecke, 2021), REdaS (Maier, 2015), sem-Tools (Jorgensen et al., 2021), semPlot (Epskamp, 2019), and equaltestMI (Jiang et al., 2021).

## Results

The descriptive analysis of each item of the Identity Distress Scale (IDS) revealed that items I4 and I5 exhibit a right-skewed distribution. Furthermore, items I4, I5, and I7 reflect a low level of difficulty. Additionally, it is shown that all items discriminate effectively *(See Table 1)*.

The KMO index = .8438 and the significant result derived from Bartlett’s sphericity test (χ^2^= 1089.355, df = 45, p < .001) indicate that the correlation matrix can be factored.

**Table 1:** Descriptive Analysis of the Variables

Items	Mean	SD	Skew	Difficulty	Discrimination Item	deleted α if
I1.	3.13	.93	–.07	.63	.51	.77
I2.	2.89	1.14	–.07	.58	.44	.77
I3.	2.64	1.01	.09	.53	.49	.77
I4.	1.53	.89	1.65	.31	.38	.78
I5.	1.64	.98	1.49	.33	.37	.78
I6.	2.19	1.07	.7	.44	.48	.77
I7.	1.94	1.1	.9	.39	.41	.78
I8.	2.34	.99	.31	.47	.54	.76
I9.	2.8	.94	.15	.56	.57	.76
I1.	2.3	1.34	.77	.46	.44	.78

The Very Simple Structure (VSS) analysis was conducted to evaluate the appropriateness of different factor solutions. According to the VSS complexity 1 and complexity 2 indices, the maximum VSS score was achieved with one factor (.68) under complexity 1 which improved to .75 with two factors under complexity 2. This indicates an incremental benefit in explanatory power with the addition of a second factor. Additionally, the Velicer MAP test supported the one-factor model, achieving a minimum score of .02, which indicates that a single factor is sufficient in capturing the underlying structure of the dataset.

The exploratory factor analysis (EFA) using unweighted least squares (ULS) estimator and oblimin rotation was performed for a two-factor solution. Both factors cumulatively accounted for 41% of the variance among the items. For factor 1, we observed high loadings on D4 (.59), D6 (.68), D8 (.63), D9 (.71), and D10 (.56), indicating a robust alignment with constructs expected to measure aspects of identity distress. Factor 2 primarily captured substantial loadings with D2 (.85) and moderate associations with D1 (.52) and D7 (.43). Most items demonstrated a high level of shared variance with their respective factors, ranging from .2 to .52 (*See Table 2*).

**Table 2:** A two-factor solution obtained through EFA

	factor 1	factor 2	communality item
D1	.24	.52	.45
D2	-.07	.85	.68
D3	.31	.37	.35
D4	.59	-.04	.33
D5	.51	.01	.26
D6	.68	-.07	.42
D7	.2	.43	.31
D8	.63	.08	.45
D9	.71	.02	.52
D10	.56	.04	.33

For confirmatory factor analysis and internal consistency analysis, a first-order two-factor model was evaluated, grouping the first 7 items into the distress factor and items 8, 9, and 10 into the global factor. The results are shown in *Table 3*.

**Table 3:** Goodness of fit and internal consistency of the two-factor model

Factor	χ^2^	df	CFI	TLI	SRMR	RM SEA	RMSEA 90% CI	α	ω	AVE
Distress	125.03	34	.97	.95	.07	.07	.06 – .09	.76	.73	.33
Global								.73	.66	.48

*Note: α, Cronbach’s alpha; ω, omega; AVE, average variance extracted*

**Figure 1.**
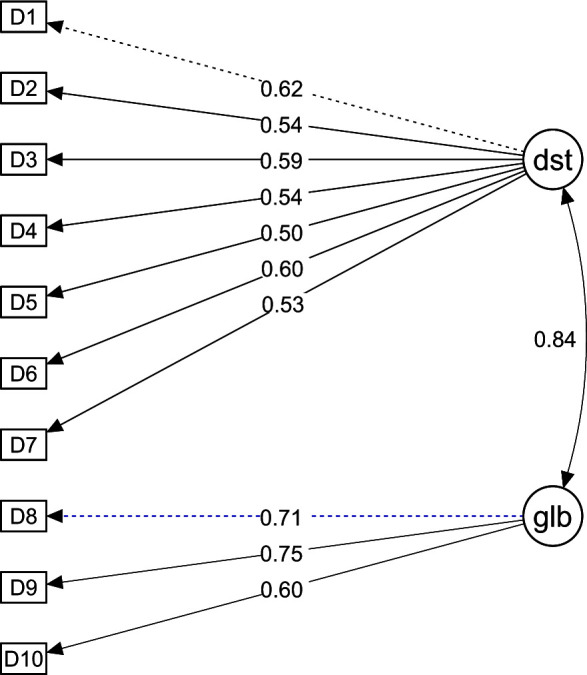
Factor loadings obtained in the confirmatory factor analysis

The first-order two-factor model yielded acceptable goodness-of-fit indices in incremental fit indices: Comparative Fit Index (CFI), Tucker-Lewis Index (TLI), and Standardized Root Mean Square Residual (SRMR). According to Hu & Bentler (1999), CFIand TLI values should be close to .95, and SRMR should be less than .08 for adequate goodness of fit. However, the Root Mean Square Error of Approximation (RMSEA) with a score of .07 and a confidence interval between .06 and .09 exceeds the expected value of .06 proposed by Hu & Bentler (1999), although other authors suggest a value of .08 (Browne & Cudeck, 1989), which still allows for a reasonable fit. *Figure 1* shows a good level of item-factor and inter-factor relationships, as the acceptable range is between .5 and 1. Cronbach’s alpha for the whole scale was .79.

The adjustment indices for calculating factorial invariance in the first-order two-factor model (for each gender separately) are displayed in *Table 4*. Configurational invariance, metric invariance, scalar invariance, and strict invariance were progressively evaluated from the established model (Byrne, 2008).

**Table 4:** Invariance analysis

Model	χ^2^	gl	CFI	RM SEA	SRMR	ΔCFI	ΔRMSEA	Δ	SRMR
Model (two groups)	14.11	34	.97	.08	.07				
Men Model	74.88	34	.97	.09	.09				
Women Model	96.61	34	.97	.073	.07				
Configural Model	171.49	68	.97	.08	.08				
Metric Model	203.01	76	.96	.08	.08	–.007	.004		.004
Scalar Model	205.74	103	.97	.06	.08	.007	–.019		–.003
Strict Model	212.82	104	.97	.06	.08	–.002	.002		0
Variance covariance and Model	222.662	107	.97	.07	.07	–.002	.001		.001

Firstly, it is observed that there is a better fit in the women’s group than in the men’s group, especially when comparing the RMSEA values. Secondly, when comparing the changes (ΔCFIand ΔRMSEA) between models, no significant changes are observed.

**Figure 2.**
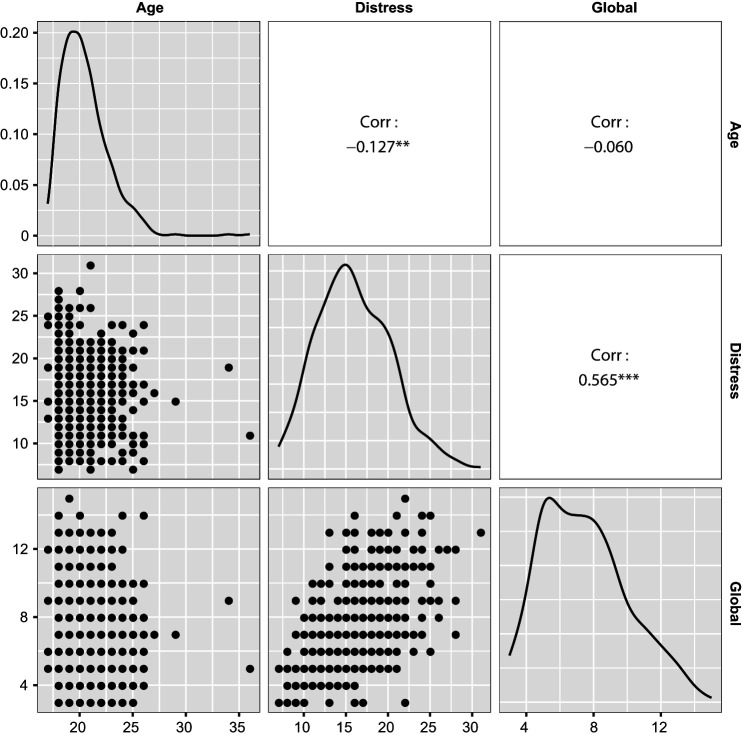
Correlations of Age with Identity Distress and Global scores of the IDS

The correlation analysis between the distress, global factors, and the age variable (see *Figure 2*) indicated that age has a low-magnitude negative relationship with the distress factor (ρ = –.13). In contrast, age does not present a relationship with the global factor (ρ = –.06). However, a moderate to high-magnitude positive relationship was observed between the distress and global factors (ρ = .57).

Furthermore, distribution tests were conducted to verify the difference between men and women. When comparing the sex category within the distress factor, it was found that there is no significant difference between men and women (*p* = .06), indicating a minimal effect (Cohen’s *d* = .18). Similarly, when comparing sex with the global factor, a similar result was obtained (*p* = .82; Cohen’s *d* = .02), indicating no significant difference between men and women.

**Figure 3.**
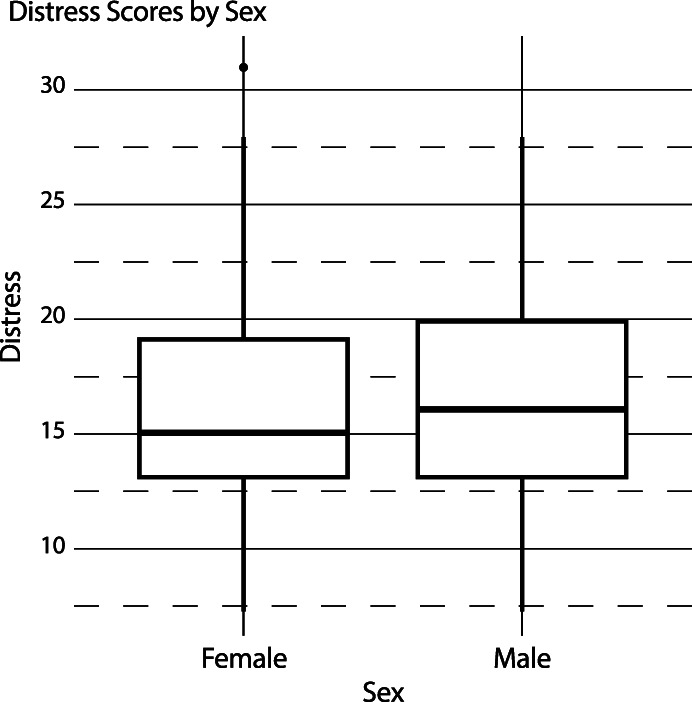
Box plot, Distress, and Sex

Lastly, when graphically comparing the behavior of the sex variable in the distress and global factors, it is observed that the distress factor in women has an atypical score and less dispersion when compared to men. Additionally, both sexes exhibit the same limits and a positive skew towards the right, although in women, it denotes a greater skew (see *Figure 3*).

**Figure 4.**
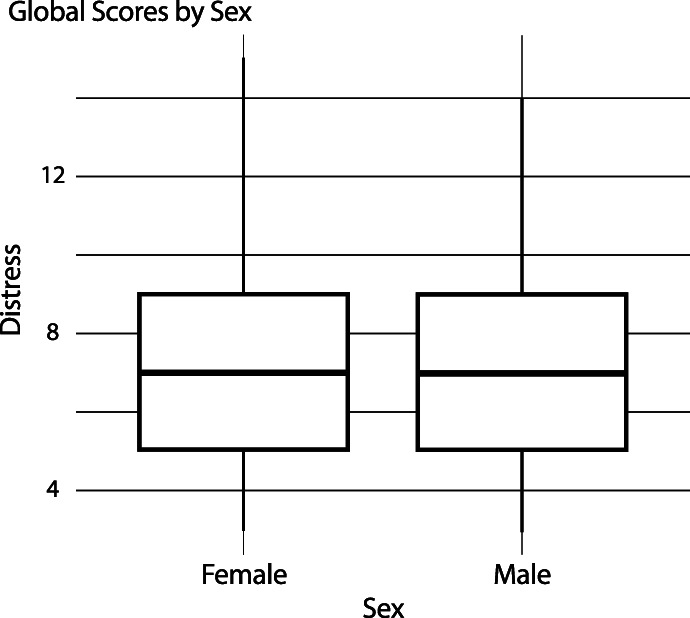
Box plot, Global, and Sex

Conversely, in the global factor, both women and men show similar dispersion. In both cases, there is a positive skew towards the right, with women showing a greater skew (see *Figure 4*).

## Discussion

The present study highlights that there are cultural factors among Ecuadorian participants may influence identity distress, potentially differing from results in other studies conducted predominantly in the Global North. This investigation found no significant differences between genders, regarding identity distress, though it acknowledged that cultural expectations concerning gender roles may still influence how individuals experience their identity issues. Moreover, emerging adulthood in Ecuador and in Latin America is shaped by unique socio-cultural factors, like familial expectations, familial closeness, economic challenges, and societal norms, which influence identity formation (Torres et al., 2023). Finally, Ecuadorian emerging adults often navigate collective cultural values that prioritize family and their community, which may impact their identity development as well as the experience of identity distress.

A deeper understanding of the development of identity, the challenges it entails, and its correlation with socio-emotional issues is essential. A thorough theoretical understanding can aid in the timely detection of these issues while enhancing their prevention and intervention (Potterton et al., 2022). Building upon this premise, the current study aimed to evaluate the IDS instrument to determine its applicability within the Ecuadorian context.

Upon evaluating the ten questions of the IDS, acceptable results were obtained. However, items I4 and I5 stand out for their right-skewed asymmetry. Similarly, items I4, I5, and I7 reflect a low level of difficulty. Possible reasons for the difficulties in each item include the significant influence of social context. As noted by Jensen (2021), culture is a core element of psychological and social processes. The church’s influence on sexual regulation (Wolf & Platt, 2022) helps explain behaviors related to item I4, particularly among women who have received restrictive sexual education (Ordoñez et al., 2017). For item I5, young people often adopt their family’s religion without critically considering personal choice (Kelemen et al., 2021), which fosters an extrinsic approach to religion that may negatively impact psychological well-being (Allport & Ross, 1967). Regarding item I7, its interpretation varies due to the negative connotation of the term *gang* in the Ecuadorian context, leading participants to distance themselves from related stress.

All items demonstrate effective discrimination, suggesting that preliminary, the scale could be used in the Ecuadorian context. These findings align with the analysis conducted by Papazova & Bakracheva (2021), who successfully adapted the IDS for the Bulgarian population, as analytical studies demonstrated the instrument’s reliability and validity for use in that cultural context.

The EFA results suggest partial confirmation of the theoretical constructs, with some notable discrepancies that may warrant model revision or further investigation. Specifically, the strong loadings of items D8, D9, and D10 on Factor 1, along with items theoretically associated with the distress factor, challenge the distinctiveness of the *global construct* as originally theorized. This might indicate that the defined constructs share more common variance than expected. Future studies might explore the inclusion of additional items or the modification of existing items.

Additionally, the present study employed the first-order two-factor model, where the distress factor was organized around items 1–7, while the global factor comprised items 8, 9, and 1. Results from this analysis demonstrate acceptable fit indices in both the CFIand TLI, as well as in the SRMR. However, the RMSEA score of .07 exceeds the expected value of .06 (Hu & Bentler, 1999), although other authors suggest .08 (Browne & Cudeck, 1989), which permits its fit. Furthermore, internal consistency values (α and ω) are considered valid when they fall between .70 and .90 (Campo-Arias & Oviedo, 2008); they reveal acceptability in the distress factor but omega fall below the acceptable range in the global factor, considering that these items have different Likert scales, particularly item 1. Findings from Janowicz et al. (2024) are consistent with the present study, indicating that the two-factor model is the optimal framework for analyzing the IDS instrument. However, it is uncertain if the original work included any factor analysis. Berman et al. (2011), in their U.S, Chinese, Japanese, and Taiwanese versions of the IDS, tried a one-factor model; however, the analyses were incomplete due to the absence of calculation for additional fit statistics (Janowicz, 2024).

It is worth noting that within the invariance analysis, the scalar invariance exhibits the most disparate values in terms of ΔRMSEA. However, this does not compromise meeting the criteria, as Rutkowski & Svetina (2014) argue that the while stricter criteria recommend ΔRMSEA values below .01, their findings suport a criterion of less than .03. This permits a proper fit, affirming that the instrument demonstrates invariance. Regarding the correlation analysis, the correlation between age and distress factor is significant but very low, whereas the relationship between age and the global factor is non-significant. It is also noteworthy that the correlation between the raw scores of the factors are moderate to high (ρ = .57). Finally, when analyzing the variable sex, both through distribution tests and graphical representations, no significant differences are observed. According to Palmeroni et al. (2019), gender differences in identity distress remain ambiguous. Current literature that addresses differences between men and women regarding identity distress is scarce, and findings remain inconsistent.

Janowicz et al. (2024), consider that analyzing this model enables the capture of the two aspects of identity distress: the intensity of the various identity domains and their interference in the individual’s daily life.

Finally, the difficulties encountered in the analysis of the items can be addressed in future research endeavors, with a focus on examining various themes such as gender, religion, and group loyalty, among others. However, such analysis was beyond the scope of the present study.

## Conclusion

The present research conducted an exploratory and confirmatory factor analysis on the Identity Distress Scale (IDS) within the Ecuadorian context. This process involved several phases aimed at determining the construct validity and internal consistency of the measurement tool. Initially, a qualitative review of the items was conducted to determine the appropriateness of the translation. Subsequently, a descriptive correlation analysis was performed for each item to investigating the levels of correlation between them. Following this, an exploratory factor analysis was executed, with the data processed under the premise of two underlying factors to which the variables correspond. Finally, in the confirmatory factor analysis, the obtained data were examined to determine their alignment with the proposed statistical model by assessing the goodness of fit and how well the data corresponded with the theoretical framework.

Throughout the various phases of this study, it became increasingly evident that the association between the latent construct and the manifest variables was strong. However, certain items (2, 4, 5, 7) exhibited statistically different behavior, not fully reflecting the identity distress construct as expected, albeit within acceptable limits. Based on the results, it is concluded that the tool demonstrates satisfactory construct validity and internal consistency, making it a reliable instrument for psychologists, researchers, educators, and students interested in studying identity distress within the Ecuadorian context.

It is recommended to expand the validation of the instrument through processes of convergent and discriminant validity. Additionally, employing other qualitative methods, such as cognitive interviews, would help understand the invariance across genders.

Furthermore, it is also suggested to expand the research by conducting studies that explore how cultural differences influence the experience of identity distress. This could include comparisons between various regions of Ecuador or among different ethnic groups. Additionally, it is recommended that the scale be applied to other population groups, encompassing different age ranges, as well as populations in migratory contexts or those that present specific implications for identity, such as women in the perinatal stage, to assess its validity and utility in diverse sociocultural contexts.

Moreover, it would be highly beneficial to incorporate qualitative methods, such as interviews or focus groups, to enrich the understanding of how individuals experience and manage identity distress, providing a deeper context to the quantitative findings.

## Limitations

The generalization of the results might be limited due to the higher prevalence of women in the study sample. Additionally, the difficulties in obtaining a larger sample, due to various factors not related to the authors, may further restrict the broader applicability of the findings.
